# (*E*)-2-[(2-Hydr­oxy-5-nitro­phen­yl)iminiometh­yl]phenolate

**DOI:** 10.1107/S1600536809001007

**Published:** 2009-02-11

**Authors:** Yousef M. Hijji, Belygona Barare, Ray J. Butcher, Jerry P. Jasinski

**Affiliations:** a Department of Chemistry, Morgan State University, Baltimore, MD 21251, USA; b Department of Chemistry, Howard University, 525 College Street NW, Washington DC 20059, USA; c Department of Chemistry, Keene State College, 229 Main Street, Keene, NH 03435-2001, USA

## Abstract

In the title mol­ecule, C_13_H_10_N_2_O_4_, the dihedral angle between the mean planes of the benzene and phenolate rings is 21.6 (4)°. The nitro O atoms are twisted slightly out of the plane of the ring to which the nitro group is attached [dihedral angle 8.4 (3)°]. The amine group forms an intra­molecular hydrogen bond with both nearby O atoms. An extended π delocalization throughout the entire mol­ecule exists producing a zwitterionic effect in this region of the mol­ecule. The shortened C—O bond [1.2997 (15) Å] in concert with the slightly longer C—OH bond [1.3310 (16) Å] provide evidence for this effect. The crystal packing is influenced by strong inter­molecular O—H⋯O hydrogen bonding. As a result, mol­ecules are linked into an infinite zigzag chain running along the *b* axis. A *MOPAC* PM3 calculation provides support to these observations.

## Related literature

For related structures, see: Ersanlı *et al.* (2003 ▶); Odabaşoğlu *et al.* (2006 ▶); Jasinski *et al.* (2007 ▶); Elerman *et al.* (1995 ▶); Hijji *et al.* (2008 ▶, 2009 ▶). For the application of Schiff bases in organic synthesis, see: Barba *et al.* (2001 ▶); Rodriguez *et al.* (2005 ▶). For details of the *MOPAC* PM3 calculation, see: Schmidt & Polik (2007 ▶).
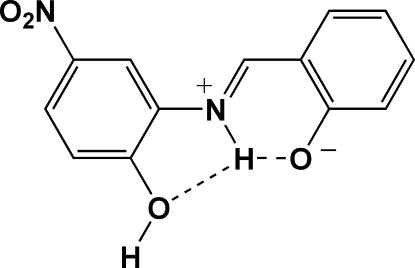


## Experimental

### 

#### Crystal data

C_13_H_10_N_2_O_4_
*M* *_r_* = 258.23
Monoclinic, 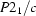

*a* = 7.3949 (3) Å
*b* = 9.1058 (4) Å
*c* = 17.2734 (6) Å
β = 96.387 (4)°
*V* = 1155.91 (8) Å^3^
*Z* = 4
Mo *K*α radiation
μ = 0.11 mm^−1^
*T* = 296 (2) K
0.45 × 0.36 × 0.21 mm

#### Data collection

Oxford Diffraction Gemini R diffractometer
Absorption correction: multi-scan (*CrysAlis RED*; Oxford Diffraction, 2007 ▶) *T*_min_ = 0.950, *T*_max_ = 0.977
16362 measured reflections
3889 independent reflections
2071 reflections with *I* > 2σ(*I*)
*R*_int_ = 0.045

#### Refinement

*R*[*F* ^2^ > 2σ(*F* ^2^)] = 0.052
*wR*(*F* ^2^) = 0.161
*S* = 1.01
3889 reflections
173 parameters
H-atom parameters constrained
Δρ_max_ = 0.36 e Å^−3^
Δρ_min_ = −0.23 e Å^−3^

### 

Data collection: *CrysAlisPro* (Oxford Diffraction, 2007 ▶); cell refinement: *CrysAlisPro*; data reduction: *CrysAlisRed* (Oxford Diffraction, 2007 ▶); program(s) used to solve structure: *SHELXS97* (Sheldrick, 2008 ▶); program(s) used to refine structure: *SHELXL97* (Sheldrick, 2008 ▶); molecular graphics: *SHELXTL* (Sheldrick, 2008 ▶); software used to prepare material for publication: *SHELXTL*.

## Supplementary Material

Crystal structure: contains datablocks global, I. DOI: 10.1107/S1600536809001007/bt2849sup1.cif

Structure factors: contains datablocks I. DOI: 10.1107/S1600536809001007/bt2849Isup2.hkl

Additional supplementary materials:  crystallographic information; 3D view; checkCIF report

## Figures and Tables

**Table 1:** Hydrogen-bond geometry (Å, °)

*D*—H⋯*A*	*D*—H	H⋯*A*	*D*⋯*A*	*D*—H⋯*A*
O1—H1*O*⋯O4^i^	0.82	1.72	2.5407 (13)	175
N2—H2*B*⋯O4	0.86	1.91	2.5973 (15)	135
N2—H2*B*⋯O1	0.86	2.34	2.6532 (14)	102
C2—H2*A*⋯O4^i^	0.93	2.60	3.2233 (18)	125
C13—H13*A*⋯O2^ii^	0.93	2.65	3.5555 (19)	163

Symmetry codes: (i) 
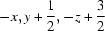
; (ii) 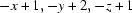.

**Fig. 1.**
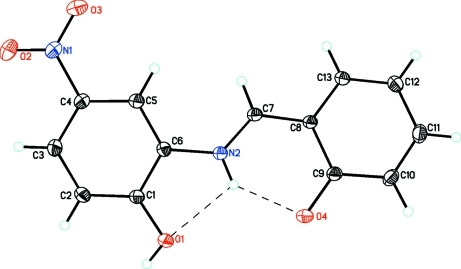
The molecular structure of C13H10N2O4, showing the atom numbering scheme and 50% probability displacement ellipsoids. Dashed lines indicate intramolecular N–H···O hydrogen bonds.

**Fig. 2.**
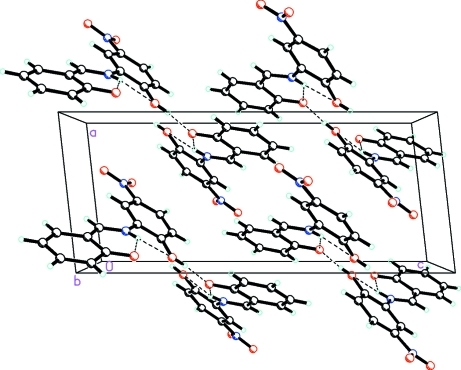
The molecular packing for C13H10N2O4 viewed down the b axis. Dashed lines indicate intermolecular O–H···O, C–H···O and intramolecular N–H···O hydrogen bonds.

## supplementary crystallographic information

### Comment

Schiff bases have extensive application in industry. Much interest in these compounds and their complexes is due to their anti-tumor activities. The boronate complex derivative of the title compound (Barba *et al.* 2001) displays it in the phenol-imine form, which can undergo an imine Diels-Alder reaction to produce a 3,4-dihydroquinoline (Rodriguez *et al.*, 2005). Compounds of this type can also be used as anion sensors in acetonitrile (Hijji *et al.*, 2008) and tend to exist in the keto-amine form, which is generally favored over the phenol-imine form in the solid state. While the keto-amine tautomer is commonly produced in these complexes (Ersanlı  *et al.*, 2003; Odabaşoğlu *et al.*, 2006; Elerman *et al.*, 1995; Jasinski *et al.*, 2007), the title compound adapts a phenol-iminio tautomer as the stable form which is confirmed by the X-ray data and in agreement with that found *via* NMR data in DMSO solution. The stability of this form may be enhanced by the electronic effect of the nitro group. The dark color in the solid state is generally an indication of increased conjugation, while the yellow colored solutions, as in acetonitrile or anhydrous DMSO, may be due to conversion to the phenol-iminio tautomer.

The title molecule, C_13_H_10_N_2_O_4_, consists of a 2-hydroxy-5-nitrophenyliminio group and a phenolate group bonded to a methylene carbon atom with both of the planar six-membered rings twisted from the plane of the molecule. The dihedral angle between the mean planes of the phenyl and phenolate rings measures 21.6 (4)°. The nitro oxygen atoms are twisted slightly out of the plane of the molecule [torsion angles = 170.80 (13) (O2—N1—C4—C5); -7.5 (2)° (O3—N1—C4—C5); -5.8 (2)° (O2—N1—C4—C3); 175.92 (14)° (O3—N1—C4—C3)]. The phenolate (O4) and hydroxy (O1) oxygen atoms are essentially in the plane of the molecule [torsion angles = -179.51 (13)° (O4—C9—C10—C11); -3.2 (2)° (O4—C9—C8—C7); -178.63 (14)° (O1—C1—C2—C3); 178.83 (12)° (O1—C1—C6—C5)]. The iminio group forms an intramolecular hydrogen bond with each of the nearby oxygen atoms (O1 and O4) (see Fig. 1 and Table 1). There appears to be an extended π delocalization effect throughout the entire molecule producing a zwitterionic effect in this region of the molecule similar to that seen in a close structurally related dinitro compound (Hijji *et al.*, 2009). The shortened C9—O4 bond (1.2997 (15) Å in concert with the slightly longer C1—O1 bond (1.3310 (16) Å) provide structural evidence of this effect in a similar fashion.

Crystal packing is influenced by extensive strong intermolecular O—H···O hydrogen bonding between the phenolate and hydroxy oxygen atoms (O4 & O1) and their respective hydrogen atoms within the π delocalized region (O1—H10···O4; 2.540 (7) Å) of the molecule. Additional weak intermolecular C—H···O hydrogen bond interactions occur involving the phenyl (C2) and phenolate (C13) groups, respectively. All of the hydrogen bond interactions are summarized in Table 1. A s a result the molecules are linked into an infinite polymeric chain diagonally along the [101] plane of the unit cell in an alternate inverted pattern (Fig. 2). In addition, weak *Cg*2–*Cg*2 (3.895 (2) Å; slippage = 1.09 (2)°; *-x, 2 - y, -z*) π-π stacking ring interactions and N1–O2···*Cg*2 π-ring interactions (3.648 (4) Å; *x, -1 + y, z*) also occur where *Cg*2 = center of gravity of the C8–C13 ring.

After a *MOPAC* PM3 calculation [Parameterized Model 3 approximation together with the Hartree-Fock closed-shell (restricted) wavefunction was used and minimizations were teminnated at an r.m.s. gradient of less than 0.01 kJ mol^-1^ Å^-1^] of the zwitterionic form with *WebMO Pro* (Schmidt & Polik, 2007), the mean planes between the phenyl and phenolate rings changes to 3.7 (7)°, producing a significantly less twisted, nearly planar, molecule than that observed in the crystalline environment. It is apparent that the extensive hydrogen bonding, π-π stacking and π-ring intermolecular interactions significantly influence crystal packing with this molecule.

### Experimental

2-Amino-4-nitrophenol (0.15 g, 1 mmol) and salicylaldehyde (0.12 g, 1 mmol) were mixed neat in a loosely capped vial, forming a light orange solid. The mixture was heated at full power in a conventional spacemaker II microwave oven for two minutes forming a deep orange product. The reaction mixture was recrystallized from a 1:1 mixture (v:v) of methanol and diethyl ether affording 0.18 g, 67% yield of the title compound as dark purple crystals. The physical data is in agreement with literature (Barba *et al.*, 2001), mp 505–508 K, GC/MS, m/*z*: 258 (*M*^+^), 211,165, 77, 51. ^1^H-NMR (400 MHz, DMSO-d6) δ (p.p.m.): 13.21(s, 1H), 11.41(s, 1H), 9.10(s, 1H), 8.28(d, J = 2.7 Hz, 1H), 8.07 (dd, J = 8.87, 2.7 Hz, 1H), 7.70 (dd, J = 7.65, 1.3 Hz, 1H), 7.44 (dt, J = 7.6,1.5 Hz, 1H), 7.14 (d, J = 9.03 Hz, 1H), 6.98 (t, J = 7.7, H), 6.96 (d, J = 8.5 Hz, 1H). ^13^C-NMR (100 MHz, DMSO-d_6_) δ (p.p.m.): 164.4 (CH), 160.5 (C), 157.6 (C), 139.9 (C), 135.7 (C), 133.5 (CH), 132.7 (CH), 123.7 (CH), 119.4 (CH), 117.2 (CH), 116.7 (CH), 116.3 (CH), 115.3 (CH).

### Refinement

All H atoms could be seen in a difference fourier map. Nevertheless, they were placed in their calculated positions and then refined using the riding model with O—H = 0.82, N—H = 0.86 and C—H = 0.93 Å, and with *U*_iso_(H) = 1.18–1.20*U*_eq_(C, N, O).

### Crystal data

**Table:**

C_13_H_10_N_2_O_4_	*F*(000) = 536
*M**_r_* = 258.23	*D*_x_ = 1.484 Mg m^−^^3^
Monoclinic, *P*2_1_/*c*	Mo *K*α radiation, λ = 0.71073 Å
Hall symbol: -P 2ybc	Cell parameters from 5321 reflections
*a* = 7.3949 (3) Å	θ = 4.6–32.6°
*b* = 9.1058 (4) Å	µ = 0.11 mm^−^^1^
*c* = 17.2734 (6) Å	*T* = 296 K
β = 96.387 (4)°	Prism, yellow
*V* = 1155.91 (8) Å^3^	0.45 × 0.36 × 0.21 mm
*Z* = 4	

### Data collection

**Table:**

Oxford Diffraction Gemini R diffractometer	3889 independent reflections
Radiation source: fine-focus sealed tube	2071 reflections with *I* > 2σ(*I*)
graphite	*R*_int_ = 0.045
Detector resolution: 10.5081 pixels mm^-1^	θ_max_ = 32.5°, θ_min_ = 4.6°
φ and ω scans	*h* = −10→11
Absorption correction: multi-scan (*CrysAlis RED*; Oxford Diffraction, 2007)	*k* = −12→13
*T*_min_ = 0.950, *T*_max_ = 0.977	*l* = −25→24
16362 measured reflections	

### Refinement

**Table:**

Refinement on *F*^2^	Primary atom site location: structure-invariant direct methods
Least-squares matrix: full	Secondary atom site location: difference Fourier map
*R*[*F*^2^ > 2σ(*F*^2^)] = 0.052	Hydrogen site location: inferred from neighbouring sites
*wR*(*F*^2^) = 0.161	H-atom parameters constrained
*S* = 1.01	*w* = 1/[σ^2^(*F*_o_^2^) + (0.0893*P*)^2^] where *P* = (*F*_o_^2^ + 2*F*_c_^2^)/3
3889 reflections	(Δ/σ)_max_ < 0.001
173 parameters	Δρ_max_ = 0.36 e Å^−^^3^
0 restraints	Δρ_min_ = −0.23 e Å^−^^3^

### Special details

**Table:**

Geometry. All e.s.d.'s (except the e.s.d. in the dihedral angle between two l.s. planes) are estimated using the full covariance matrix. The cell e.s.d.'s are taken into account individually in the estimation of e.s.d.'s in distances, angles and torsion angles; correlations between e.s.d.'s in cell parameters are only used when they are defined by crystal symmetry. An approximate (isotropic) treatment of cell e.s.d.'s is used for estimating e.s.d.'s involving l.s. planes.
Refinement. Refinement of *F*^2^ against ALL reflections. The weighted *R*-factor *wR* and goodness of fit *S* are based on *F*^2^, conventional *R*-factors *R* are based on *F*, with *F* set to zero for negative *F*^2^. The threshold expression of *F*^2^ > σ(*F*^2^) is used only for calculating *R*-factors(gt) *etc*. and is not relevant to the choice of reflections for refinement. *R*-factors based on *F*^2^ are statistically about twice as large as those based on *F*, and *R*- factors based on ALL data will be even larger.

### Fractional atomic coordinates and isotropic or equivalent isotropic displacement parameters (Å^2^)

**Table:**

	*x*	*y*	*z*	*U*_iso_*/*U*_eq_	
O1	0.07674 (16)	0.78106 (11)	0.74658 (6)	0.0506 (3)	
H1O	0.0213	0.8253	0.7779	0.061*	
O2	0.56661 (19)	1.27359 (14)	0.63179 (8)	0.0696 (4)	
O3	0.64896 (19)	1.09097 (16)	0.56567 (8)	0.0797 (5)	
O4	0.10989 (15)	0.42056 (11)	0.66269 (5)	0.0475 (3)	
N1	0.56076 (17)	1.14379 (16)	0.61491 (7)	0.0494 (3)	
N2	0.25299 (15)	0.67241 (13)	0.63378 (6)	0.0367 (3)	
H2B	0.2098	0.6146	0.6665	0.044*	
C7	0.27637 (17)	0.61689 (15)	0.56560 (7)	0.0353 (3)	
H7A	0.3301	0.6755	0.5304	0.042*	
C1	0.19513 (18)	0.87143 (15)	0.71860 (7)	0.0372 (3)	
C2	0.2271 (2)	1.01473 (16)	0.74472 (8)	0.0433 (4)	
H2A	0.1653	1.0508	0.7847	0.052*	
C3	0.3485 (2)	1.10366 (16)	0.71228 (8)	0.0430 (4)	
H3A	0.3688	1.1995	0.7297	0.052*	
C4	0.44018 (19)	1.04784 (16)	0.65308 (8)	0.0383 (3)	
C5	0.41405 (19)	0.90591 (15)	0.62591 (8)	0.0382 (3)	
H5A	0.4778	0.8706	0.5863	0.046*	
C6	0.29168 (18)	0.81772 (15)	0.65867 (7)	0.0346 (3)	
C8	0.22475 (18)	0.47420 (15)	0.54298 (7)	0.0330 (3)	
C9	0.13664 (18)	0.37863 (15)	0.59295 (7)	0.0357 (3)	
C10	0.0795 (2)	0.23976 (16)	0.56283 (8)	0.0430 (4)	
H10A	0.0199	0.1758	0.5934	0.052*	
C11	0.1105 (2)	0.19794 (16)	0.48953 (9)	0.0464 (4)	
H11A	0.0724	0.1056	0.4714	0.056*	
C12	0.1984 (2)	0.29107 (17)	0.44103 (8)	0.0443 (4)	
H12A	0.2196	0.2601	0.3915	0.053*	
C13	0.25243 (19)	0.42681 (15)	0.46681 (8)	0.0380 (3)	
H13A	0.3082	0.4895	0.4342	0.046*	

### Atomic displacement parameters (Å^2^)

**Table:**

	*U*^11^	*U*^22^	*U*^33^	*U*^12^	*U*^13^	*U*^23^
O1	0.0648 (7)	0.0424 (6)	0.0510 (6)	−0.0085 (5)	0.0346 (5)	−0.0106 (5)
O2	0.0823 (9)	0.0445 (8)	0.0842 (9)	−0.0225 (7)	0.0183 (7)	−0.0007 (6)
O3	0.0888 (10)	0.0751 (10)	0.0847 (9)	−0.0308 (8)	0.0520 (8)	−0.0116 (7)
O4	0.0642 (7)	0.0413 (6)	0.0418 (5)	−0.0029 (5)	0.0272 (5)	0.0021 (4)
N1	0.0507 (8)	0.0468 (8)	0.0520 (7)	−0.0143 (6)	0.0115 (6)	0.0001 (6)
N2	0.0446 (6)	0.0301 (6)	0.0385 (6)	−0.0019 (5)	0.0180 (5)	−0.0009 (5)
C7	0.0367 (7)	0.0335 (7)	0.0383 (7)	−0.0011 (6)	0.0153 (5)	0.0007 (5)
C1	0.0465 (8)	0.0325 (7)	0.0348 (6)	−0.0015 (6)	0.0140 (6)	0.0004 (5)
C2	0.0530 (9)	0.0387 (8)	0.0407 (7)	0.0013 (7)	0.0159 (6)	−0.0064 (6)
C3	0.0518 (9)	0.0337 (8)	0.0439 (7)	−0.0056 (6)	0.0068 (6)	−0.0033 (6)
C4	0.0412 (7)	0.0361 (8)	0.0383 (7)	−0.0059 (6)	0.0075 (6)	0.0018 (6)
C5	0.0422 (8)	0.0354 (8)	0.0390 (7)	−0.0021 (6)	0.0138 (6)	−0.0021 (6)
C6	0.0407 (7)	0.0298 (7)	0.0346 (6)	0.0003 (6)	0.0091 (5)	−0.0021 (5)
C8	0.0347 (6)	0.0303 (7)	0.0360 (6)	0.0010 (5)	0.0133 (5)	0.0007 (5)
C9	0.0378 (7)	0.0324 (7)	0.0390 (7)	0.0034 (6)	0.0138 (5)	0.0035 (5)
C10	0.0466 (8)	0.0327 (8)	0.0509 (8)	−0.0040 (6)	0.0109 (6)	0.0070 (6)
C11	0.0552 (9)	0.0316 (8)	0.0522 (8)	−0.0028 (7)	0.0050 (7)	−0.0038 (6)
C12	0.0551 (9)	0.0401 (9)	0.0390 (7)	0.0018 (7)	0.0113 (6)	−0.0043 (6)
C13	0.0434 (7)	0.0347 (8)	0.0382 (7)	0.0007 (6)	0.0156 (6)	0.0002 (5)

### Geometric parameters (Å, °)

**Table:**

O1—C1	1.3310 (16)	C3—C4	1.3847 (19)
O1—H1O	0.8200	C3—H3A	0.9300
O2—N1	1.2170 (18)	C4—C5	1.3813 (19)
O3—N1	1.2262 (16)	C5—C6	1.3776 (19)
O4—C9	1.2997 (15)	C5—H5A	0.9300
N1—C4	1.4574 (18)	C8—C13	1.4210 (17)
N2—C7	1.3105 (16)	C8—C9	1.4325 (18)
N2—C6	1.4107 (17)	C9—C10	1.414 (2)
N2—H2B	0.8600	C10—C11	1.366 (2)
C7—C8	1.3977 (19)	C10—H10A	0.9300
C7—H7A	0.9300	C11—C12	1.402 (2)
C1—C2	1.392 (2)	C11—H11A	0.9300
C1—C6	1.4089 (18)	C12—C13	1.359 (2)
C2—C3	1.374 (2)	C12—H12A	0.9300
C2—H2A	0.9300	C13—H13A	0.9300
			
C1—O1—H1O	109.5	C6—C5—H5A	120.7
O2—N1—O3	122.66 (14)	C4—C5—H5A	120.7
O2—N1—C4	118.75 (13)	C5—C6—C1	120.61 (12)
O3—N1—C4	118.56 (14)	C5—C6—N2	122.80 (12)
C7—N2—C6	126.38 (12)	C1—C6—N2	116.58 (12)
C7—N2—H2B	116.8	C7—C8—C13	118.57 (12)
C6—N2—H2B	116.8	C7—C8—C9	121.65 (11)
N2—C7—C8	123.41 (12)	C13—C8—C9	119.70 (12)
N2—C7—H7A	118.3	O4—C9—C10	122.26 (12)
C8—C7—H7A	118.3	O4—C9—C8	120.42 (12)
O1—C1—C2	123.82 (12)	C10—C9—C8	117.32 (12)
O1—C1—C6	117.37 (12)	C11—C10—C9	121.09 (13)
C2—C1—C6	118.80 (12)	C11—C10—H10A	119.5
C3—C2—C1	121.01 (13)	C9—C10—H10A	119.5
C3—C2—H2A	119.5	C10—C11—C12	121.48 (14)
C1—C2—H2A	119.5	C10—C11—H11A	119.3
C2—C3—C4	118.72 (14)	C12—C11—H11A	119.3
C2—C3—H3A	120.6	C13—C12—C11	119.57 (13)
C4—C3—H3A	120.6	C13—C12—H12A	120.2
C5—C4—C3	122.21 (13)	C11—C12—H12A	120.2
C5—C4—N1	118.47 (13)	C12—C13—C8	120.82 (13)
C3—C4—N1	119.23 (13)	C12—C13—H13A	119.6
C6—C5—C4	118.64 (12)	C8—C13—H13A	119.6
			
C6—N2—C7—C8	−175.95 (12)	C2—C1—C6—N2	−179.38 (12)
O1—C1—C2—C3	−178.63 (14)	C7—N2—C6—C5	−22.8 (2)
C6—C1—C2—C3	0.9 (2)	C7—N2—C6—C1	155.85 (13)
C1—C2—C3—C4	−0.4 (2)	N2—C7—C8—C13	178.40 (13)
C2—C3—C4—C5	−0.3 (2)	N2—C7—C8—C9	1.8 (2)
C2—C3—C4—N1	176.22 (13)	C7—C8—C9—O4	−3.2 (2)
O2—N1—C4—C5	170.80 (13)	C13—C8—C9—O4	−179.81 (12)
O3—N1—C4—C5	−7.5 (2)	C7—C8—C9—C10	176.10 (12)
O2—N1—C4—C3	−5.8 (2)	C13—C8—C9—C10	−0.49 (19)
O3—N1—C4—C3	175.92 (14)	O4—C9—C10—C11	−179.51 (13)
C3—C4—C5—C6	0.4 (2)	C8—C9—C10—C11	1.2 (2)
N1—C4—C5—C6	−176.08 (12)	C9—C10—C11—C12	−0.6 (2)
C4—C5—C6—C1	0.1 (2)	C10—C11—C12—C13	−0.8 (2)
C4—C5—C6—N2	178.66 (12)	C11—C12—C13—C8	1.5 (2)
O1—C1—C6—C5	178.83 (12)	C7—C8—C13—C12	−177.54 (13)
C2—C1—C6—C5	−0.7 (2)	C9—C8—C13—C12	−0.8 (2)
O1—C1—C6—N2	0.15 (19)		

### Hydrogen-bond geometry (Å, °)

**Table:**

*D*—H···*A*	*D*—H	H···*A*	*D*···*A*	*D*—H···*A*
O1—H1O···O4^i^	0.82	1.72	2.5407 (13)	175
N2—H2B···O4	0.86	1.91	2.5973 (15)	135
N2—H2B···O1	0.86	2.34	2.6532 (14)	102
C2—H2A···O4^i^	0.93	2.60	3.2233 (18)	125
C13—H13A···O2^ii^	0.93	2.65	3.5555 (19)	163

Symmetry codes: (i) −*x*, *y*+1/2, −*z*+3/2; (ii) −*x*+1, −*y*+2, −*z*+1.

## References

[bb1] Barba, V., Cuahutle, D., Santillan, R. & Farfan, N. (2001). *Can. J. Chem.* **79**, 1229–1237.

[bb2] Elerman, Y., Elmali, A., Atakol, O. & Svoboda, I. (1995). *Acta Cryst.* C**51**, 2344–2346.

[bb3] Ersanlı, C. C., Albayrak, Ç., Odabaşoǧlu, M. & Erdönmez, A. (2003). *Acta Cryst.* C**59**, o601–o602. 10.1107/s0108270103018456 14532684

[bb4] Hijji, Y. M., Barare, B., Butcher, R. J. & Jasinski, J. P. (2009). *Acta Cryst.* E**65**, o291–o292. 10.1107/S1600536809000543 PMC2968345 21581902

[bb5] Hijji, Y. M., Barare, B., Kennedy, A. P. & Butcher, R. (2008). *Sensors Actuators B Chem* doi:10.1016/j.*SnB*2008.11.045.

[bb6] Jasinski, J. P., Butcher, R. J., Narayana, B., Swamy, M. T. & Yathirajan, H. S. (2007). *Acta Cryst.* E**63**, o4566–o4567.

[bb7] Odabaşoğlu, M., Albayrak, C. & Büyükgüngör, O. (2006). *Acta Cryst.* E**62**, o1094–o1096. 10.1107/S0108270106005026 16598136

[bb8] Oxford Diffraction (2007). *CrysAlisPro* and *CrysAlis RED* Oxford Diffraction Ltd, Abingdon, England.

[bb9] Rodriguez, M., Ocha, M. E., Santillan, R., Farfan, N. & Barba, V. (2005). *J. Organomet. Chem.*, **690**, 2975–2988.

[bb10] Schmidt, J. R. & Polik, W. F. (2007). *WebMO Pro* WebMO, LLC, Holland, MI, USA, available from http://www.webmo.net.

[bb11] Sheldrick, G. M. (2008). *Acta Cryst.* A**64**, 112–122. 10.1107/S0108767307043930 18156677

